# Does ‘net zero’ mean zero cows?

**DOI:** 10.1080/00963402.2024.2339068

**Published:** 2024-05-06

**Authors:** John Lynch, Raymond Pierrehumbert

**Keywords:** Climate change, livestock, methane, net zero, global warming, agriculture

## Abstract

A significant share of anthropogenic global warming comes from livestock production. There is debate about whether there can be any role for livestock in a climatically sustainable future; the debate is particularly heated for cows and sheep, largely due to the methane they burp out. However, short-lived gases like methane affect climate in a fundamentally different way than long-lived gases like carbon dioxide. Consequently, climate stabilization does not require zeroing-out cattle herds. But this doesn’t mean we can eat our beef and have it (a tolerable climate) too—livestock still contribute to global warming. Preventing or limiting future growth in livestock-related emissions can represent a sensible part of the portfolio of responses to the climate crisis, particularly when carbon dioxide emissions are not on track to reach net zero sufficiently quickly.

All sectors of the economy must contribute to climate change mitigation to meet the temperature targets outlined in the Paris Agreement. Ambition—at least in the setting of targets—is ramping up, with an increasing number of countries and companies setting goals of so-called “net zero” greenhouse gas emissions. Energy generation, the largest contributor to global warming, is expected to achieve this goal mostly through the expanded deployment of renewable technologies. While this energy transition isn’t yet reflected in reduced global fossil emissions, which continue to increase (Friedlingstein et al. 2023), it is thought to provide a feasible route to decarbonizing this major source of emissions. It is proving harder to map out how agriculture might be able to reduce emissions so dramatically.

The concept of net zero applies chiefly to carbon dioxide emissions. Because carbon dioxide, once emitted into the atmosphere, causes warming that extends over millennia even after emissions cease, any continued emission of carbon dioxide causes the gas to build up in the atmosphere, making warming increase so long as carbon emissions continue. Therefore, to halt warming, it is essential that carbon dioxide emissions be zeroed out. The “net” in “net zero” recognizes that there may be opportunities, at least to a limited extent, to offset some continued emission of carbon dioxide through actions such as reforestation or the direct removal of carbon dioxide from the atmosphere by industrial processes. There are a few other gases (e.g. sulfur hexafluoride) which have millennial-scale climate impacts, but these are minor players in comparison to carbon dioxide, due to the relative quantities emitted.

Methane is very different from carbon dioxide; though a molecule of methane contributes considerably more to climate change than a molecule of carbon dioxide, that methane molecule persists in the form of methane on a time-scale of a mere dozen years, on average. The attempt to shoehorn methane into a net zero framework has led to great confusion, perhaps nowhere more than in agricultural policy. Does the concept of “net zero” even apply to emissions of short-lived greenhouse gases? What is the correct role of the control of such gases in the context of a net zero commitment? These questions lie at the heart of the problem of formulating climate-friendly agricultural policy.

Food system greenhouse gas emissions are growing (Crippa et al. 2021), and this trend is incompatible with meeting the Paris Agreement (Clark et al. 2020). The big three greenhouse gasses associated with agriculture are carbon dioxide, methane, and nitrous oxide. Carbon dioxide emissions arise from agricultural energy use; the use of the Haber process to produce fertilizer; and deforestation; and they are subject to the same net zero requirement as any other carbon dioxide emission source. While there are possibilities to stop agriculture-related deforestation (or provide carbon sinks from ecological restoration of retired agricultural land) and to decarbonize agricultural energy uses, there are few if any quick fixes that could completely cut out the food production-related emissions of methane and nitrous oxide. Of these, methane looms large in most discussions of agricultural impacts, and we will focus our discussion in that direction.

One of the systemic changes most frequently highlighted for the reduction of methane is a reduction, or even total removal, of livestock farming—especially cattle. As climate policies ramp up, governments are starting to explore what a decrease in cattle populations might achieve, and how it could be brought about. Imminent requirements for reductions to be imposed (Dutkiewicz 2023) have been suggested. In this article, we will argue that such reductions are not essential in the context of net zero goals.

## What’s the beef with beef?

Ruminants—that is, cows, sheep and goats, alongside a few other non-domestic species—are remarkable animals. Vertebrates cannot directly digest cellulose, a complex polysaccharide and important component of plant cell walls. Ruminants, though, have a complex microbial gut flora that catalyze the breakdown of cellulose into simpler carbohydrates which the animal can digest—a process termed “enteric fermentation.” The ruminant digestive system (more specifically the reticulorumen, the first compartments of their stomachs where this microbial cellulose breakdown occurs) is anaerobic, however, and methane is generated as a byproduct of enteric fermentation. This methane is subsequently burped out by the animal (90-plus percent of ruminant methane comes out this end of the digestive system), entering the atmosphere and acting as a greenhouse gas.

And ruminants produce a lot of methane. For each kilogram of beef that you eat, the steer that produced it emitted nearly a kilogram of methane during its short life (Pierrehumbert and Eshel 2015). This methane production means that foodstuffs made from ruminants have an especially large climate impact compared to other food products, and it makes ruminant farming an important consideration in planning comprehensive climate change mitigation pathways.

A range of management and technical options may help to reduce the amount of methane produced by enteric fermentation in ruminant livestock, from breeding for low-methane animals to changes in feed composition (FAO 2023). Some of these are simply the implementation of best practices: Improved animal health, for example, means more animal feed goes into growth and milk production rather than fighting off disease, and fewer replacement animals need to be reared to maintain herd size and food output. Other, more novel interventions include altering the rumen through chemical or natural dietary additives, or vaccination against methanogenic microbes.

These methane mitigations are at different stages of development, although many are already being put into practice, and it is likely they will help reduce ruminant methane emissions in the future. More research is required before rolling out some more novel methods, to confirm their efficacy and explore whether there are wider detrimental tradeoffs—particularly if they may impact ruminant or human health. Furthermore, there are limits to the scalability of most practices depending on location and system type: It is one thing to give a daily supplement to animals in a confined feedlot, another for animals grazing on extensive rangelands.

Ultimately, even if we rapidly scale-up deployment of these measures, we do not expect it will be possible to eliminate ruminant methane.

## Net zero

If methane is an unavoidable consequence of having cattle, and if net zero emissions are interpreted as applying to all greenhouse gases (not just carbon dioxide), then we have something of a dilemma. Will we have to have zero ruminants to meet the Paris Agreement?

For carbon dioxide, the safe level of ongoing emissions is net zero: Due to the extremely long atmospheric lifetime of the gas, carbon dioxide emissions have a cumulative impact, and any continued emissions will raise temperatures further with each passing year. Recognizing this surprisingly straightforward physical fact is what led to the call for net zero to become the primary climate change mitigation objective (Allen et al. 2022a). The speed at which we reach net zero carbon dioxide governs how much we will have emitted and therefore the amount of warming the world will be plateauing at after net zero is achieved. We will need to stay at net zero carbon dioxide emissions to keep temperatures from resuming their rise.

Methane doesn’t work like this. With an average atmospheric lifetime of around a decade before methane oxidizes in the atmosphere, within decades ongoing anthropogenic methane emissions can be balanced by natural atmospheric removals in a way that cannot be achieved for chemically inert carbon dioxide (Pierrehumbert 2014). Methane emissions do cause warming, but the dynamics are fundamentally quite different from carbon dioxide, and it is possible to have some level of potentially sustainable methane emissions in a way that isn’t the case for carbon dioxide.

So if the physical requirements for, and outcomes from achieving, so-called “net zero” differ between these two gases, what does this mean for climate policy? Here, things are not entirely clear.

Greenhouse gas reporting typically utilizes “equivalent emission”—in other words, weighting factors express how much a quantity of one gas is worth relative to another. The most widespread of these, as used for most policy purposes, is the 100-year Global Warming Potential, or GWP100, which reports relative climate impacts of an individual emission averaged out over the 100 years post-release. This approach has been criticized, with warnings that it obscures the dynamics and can lead to a short-sighted emphasis on methane when carbon dioxide reductions remain more fundamentally necessary (Pierrehumbert 2014). This problem is escalated with the increasing use of GWP20, the 20-year version of the Global Warming Potential (McKenzie 2023).

The Paris Agreement refers to achieving a “balance between anthropogenic emissions by sources and removals by sinks of greenhouse gases,” but it is not entirely clear precisely what this means (Fuglestvedt et al. 2018). From a physical science perspective, it has been argued that net zero should be understood, and targets set, in terms of equivalent temperature outcomes. There are novel ways of describing different equivalent emissions to reflect this, and it would entail different requirements for longer- and shorter- lived gases to achieve the same physical outcome of temperature-stabilization (Allen et al. 2022a).

Viewed in terms of temperature targets, the essential problem with GWP100 or GWP20 is that they are “kilogram-for-kilogram” conversions. GWP100 claims that the emission of 1 kilogram of methane has the same climate effect as the emission of about 27 kilograms of carbon dioxide; for GWP20 that same kilogram translates into about 80 kilograms of carbon dioxide. Seen through the lens of either conversion factor, net zero would indeed translate into “zero cows.” However, these GWP metrics do not correctly map onto the temperature consequences of emission of a short-lived gas like methane. If we introduce a new methane emission source at a certain rate of kilograms per year, atmospheric methane concentrations will rise for about 20 years, but then stabilize as the emission rate comes into balance with natural sinks.

Most of the resulting warming will stabilize with concentration, though there is a small residual increase beyond 20 years associated with the long time it takes for the deep ocean to warm up. Consequently, for a good approximation, the introduction of a new methane source will cause a warming of a certain amount in the first 20 years, but the next 20 years will produce almost no additional warming, and so on to eternity. GWP20, in contrast, would erroneously ascribe additional warming to each subsequent 20-year chunk of emissions. This is a fatal flaw, and if taken seriously would lead to perverse policy outcomes.

The true equivalence is not between a kilogram of methane and X kilograms of carbon dioxide, but between a methane emission *rate* of one kilogram per year and a one-time emission *amount* of Y kilograms of carbon dioxide. In round numbers, it turns out that introducing a new methane source with an emission rate of 1 kilogram per year is equivalent to a one-time emission of about 1,000 kilograms of carbon (3,700 kilograms of carbon dioxide) (Pierrehumbert and Eshel 2015). To put it in personal terms, if you were to permanently reduce your beef consumption by 1 kilogram per year, it would give you license to emit an additional 1,000 kilograms of carbon over your entire lifetime. To put that in perspective, that’s about two months of carbon emissions by the average US resident. Giving up that beef makes a non-trivial contribution to climate, but it’s not something that should distract you from installing a heat pump or solar panels or getting an electric car. (It should be noted that this equivalence example only works if you live forever and keep your commitment to reduced meat consumption forever. In reality, somebody would eventually need to sign on to fulfill your commitment when you can no longer do so, and this would have to be kept up through all future time, lest the benefits of your sacrifice be undone.)

Despite different views on what net zero should mean for policy purposes, the dynamics of longer- and shorter- lived gas emission pathways on the climate are universally understood (Allen et al. 2022b). From a physical climate perspective, at least, it does reveal that there is a broader range of options in terms of potentially sustainable emission pathways for agriculture than for fossil carbon release (Lynch et al. 2021).

## A question of efficiency

Even if we take a temperature-stabilizing perspective on what net zero should mean for non-carbon dioxide gases, livestock—especially ruminants—aren’t off the hook.

The microbe-assisted digestive trick that ruminants employ to consume less digestible plant matter also makes them relatively wasteful, as the share of plant biomass converted to methane and burped out is a chunk of energy that the animal doesn’t get. Monogastrics—pigs, chickens, and humans, for example—may not be able to break down cellulose, but they can convert more digestible plants more efficiently.

This isn’t the end of the inefficiencies. Animals will always lose some of the energy and nutrient content they consume in their feed through respiration and excretion. Fundamentally, this means you have to grow more crops to produce the same amount of human-consumable food with a wasteful animal step in the middle, compared to people just consuming plants directly. More crop production entails more energy use and more agricultural inputs (fertilizers and pesticides), and so affects not just climate, but broader water quality and wildlife. These issues apply to most livestock, hence there are climate and environmental arguments for reducing consumption of all animal products, not just ruminant meat and milk.

This need to produce more crops for animal agriculture also raises perhaps the most important inefficiency: land use. Habitat conversion for agriculture is the main cause of the loss of global biodiversity; to stop the decline in biodiversity we will need to rapidly turn this around, undertaking significant ecological restoration. This would also restore natural habitat carbon stocks in vegetation and soils.

Further competition for land-use is anticipated to meet increased biomass demand in future economies. A process known as Bioenergy with Carbon Capture and Storage (BECCS) promises to displace fossil fuels in the energy supply, with the added benefit of contributing to carbon removal—although raises it other concerns for biodiversity and the environment (Smith et al. 2019). Materials currently reliant on fossil fuels may also be replaced with biomass-derived substitutes, further increasing the area required for a sustainable “bioeconomy,” as it has been called (Többen et al. 2022).

There will be some locations and farming practices where livestock can facilitate carbon storage and contribute to biodiversity protection. But the current scale of animal agriculture goes well beyond these mutually beneficial opportunities for so-called “win-win” land-sharing. There are legitimate questions about just quite how much land use by animal agriculture can be justified.

## Priorities

So, in the end, where does livestock methane emission reduction fit into a net zero strategy?

First, it is necessary to distinguish the effect of a net zero target from the effect of a carbon budget target. Mapped onto global temperature effects, the net zero goal translates into policies that stabilize temperature—i.e., keep it from continuing to rise in the future. In this context, any policy which helps to keep the methane emissions rate from increasing is compatible with net zero.

However, net zero is only part of the story. Net zero does halt further warming, but it does not determine how hot the world has gotten at the time net zero is achieved. For carbon dioxide, that is determined by the carbon budget—the aggregate amount of carbon dioxide we have dumped into the atmosphere at the time net zero is achieved.

In round numbers, considering carbon dioxide alone, a carbon budget of a trillion metric tons of carbon (3.7 trillion metric tons of carbon dioxide) corresponds to a bit under 2 degrees Celsius of warming—an amount of warming that will persist for millennia even after emissions cease.

Continued methane emissions at a fixed rate add a fixed increment of temperature to the warming caused by carbon dioxide and so lower the carbon budget compatible with a target warming limit of, say, 2 degrees Celsius. If the methane emission rate is stabilized at a high value, the allowable amount of cumulative carbon dioxide emissions compatible with a 2-degree Celsius target becomes lower, generally requiring net zero carbon dioxide emissions to be achieved more rapidly. Conversely, if the methane emission rate is reduced, a modest delay in the date for achievement of net-zero carbon dioxide emissions can become compatible with the temperature target.

To some, the broader environmental impacts and foregone opportunities—or simply the ethics—of raising and slaughtering animals for human consumption mean that there is no place for livestock production. Reducing livestock methane would also provide a valuable and feasible contribution to climate change mitigation (Reisinger et al. 2021), and the few tenths of a degree that could be shaved off could be critical to staying within temperature targets, though only in a situation in which delays in achieving net zero carbon dioxide emissions are modest. Some level of reduction in livestock numbers, especially in advanced economies, looks desirable to meet our climate and other environmental goals, though a bigger threat to climate is future growth in worldwide beef and dairy consumption as relatively poor countries become relatively richer (Pierrehumbert and Eshel 2015).

We should not fool ourselves that getting rid of animal agriculture would solve climate change, though. At best, removing cows could make up for a couple decades of continued inaction in reducing fossil emissions.

It has been suggested that slowing the required rate of energy decarbonization through dietary change can lower the costs of this transition (Bryngelsson et al. 2017), revealing an interesting tradeoff in cost-effectiveness, if not absolute emission requirements, across sectors. As the political realities of enacting mitigation policy start to hit home, more needs to be done to determine what behaviors people are willing to change, and to what degree, to explore whether delaying the energy transition really is a preferred, cost-effective option. Given our continued inaction, however, sticking to the Paris Agreement temperature limits requires us to get much more serious about energy decarbonization very quickly, whatever happens in the rest of the economy.

## References

[cit0001] Allen, M. R., P. Friedlingstein, Cécile A.J. Girardin, S. Jenkins, Y. Malhi, E. Mitchell-Larson, G. P. Peters, et al. 2022a. “Net Zero: Science, Origins and Implications.” *Annual Review of Environment and Resources* 47 (1): 849–887. 10.1146/annurev-environ-112320-105050.

[cit0002] Allen, M. R., G. P. Peters, K. P. Shine, C. Azar, P. Balcombe, O. Boucher, M. Cain, et al. 2022b. “Indicate Separate Contributions of Long-Lived and Short-Lived Greenhouse Gases in Emission Targets.” *npj Climate and Atmospheric Science* 5: 5. 10.1038/s41612-021-00226-2. 35295182 PMC7612487

[cit0003] Bryngelsson, D., F. Hedenus, D. Johansson, C. Azar, and S. Wirsenius. 2017. “How Do Dietary Choices Influence the Energy-System Cost of Stabilizing the Climate?” *Energies* 10 (2): 182. 10.3390/en10020182.

[cit0004] Clark, M. A., Nina G. G. Domingo, K. Colgan, S. K. Thakrar, D. Tilman, J. Lynch, I. L. Azevedo, et al. 2020. “Global Food System Emissions Could Preclude Achieving the 1.5° and 2°C Climate Change Targets.” *Science* 370 (6517): 705–708. 10.1126/science.aba7357. 33154139

[cit0005] Crippa, M., E. Solazzo, D. Guizzardi, F. Monforti-Ferrario, F. N. Tubiello, and A. Leip. 2021. “Food Systems Are Responsible for a Third of Global Anthropogenic GHG Emissions.” *Nature Food* 2 (3): 198–209. 10.1038/s43016-021-00225-9. 37117443

[cit0006] Dutkiewicz, J. 2023. “Ireland Isn’t Culling Cows for Climate. But Maybe it Should Be?” *Bulletin of the Atomic Scientists*. October 25. https://thebulletin.org/2023/10/ireland-isnt-culling-cows-for-climate-but-maybe-they-should-be/.

[cit0007] FAO (Food and Agriculture Organization of the United Nations). 2023. “Methane Emissions in Livestock and Rice Systems—Sources, Quantification, Mitigation and Metrics.” *Rome*. 10.4060/cc7607en.

[cit0008] Friedlingstein, P., M. O’Sullivan, M. W. Jones, R. M. Andrew, Dorothee C. E. Bakker, J. Hauck, et al. 2023. “Global Carbon Budget 2023.” *Earth System Science Data* 15 (12): 5301–5369. 10.5194/essd-15-5301-2023.

[cit0009] Fuglestvedt, J., J. Rogelj, R. J. Millar, M. Allen, O. Boucher, M. Cain, P. M. Forster, E. Kriegler, and D. Shindell. 2018. “Implications of Possible Interpretations of ‘Greenhouse Gas Balance’ in the Paris Agreement.” *Philosophical Transactions of the Royal Society A: Mathematical, Physical and Engineering Sciences* 376 (2119): 20160445. 10.1098/rsta.2016.0445. PMC5897819 29610378

[cit0010] Lynch, J., M. Cain, D. Frame, and R. Pierrehumbert. 2021. “Agriculture’s Contribution to Climate Change and Role in Mitigation Is Distinct from Predominantly Fossil CO2-Emitting Sectors.” *Frontiers in Sustainable Food Systems* 4: 518039. 10.3389/fsufs.2020.518039. 33644695 PMC7116829

[cit0011] McKenzie, J. 2023. “‘Mass Delusion and Wishful thinking’: Why Everything You Think You Know About Methane Is Probably Wrong.” *Bulletin of the Atomic Scientists*. December 18. https://thebulletin.org/2023/12/mass-delusion-and-wishful-thinking-why-everything-you-think-you-know-about-methane-is-probably-wrong/.

[cit0012] Pierrehumbert, R. T. 2014. “Short-Lived Climate Pollution.” *Annual Review of Earth and Planetary Sciences* 42 (1): 341–379. 10.1146/annurev-earth-060313-054843.

[cit0013] Pierrehumbert, R. T., and G. Eshel. 2015. “Climate Impact of Beef: An Analysis Considering Multiple Time Scales and Production Methods Without Use of Global Warming Potentials.” *Environmental Research Letters* 10 (8): 085002. 10.1088/1748-9326/10/8/085002.

[cit0014] Reisinger, A., H. Clark, A. L. Cowie, J. Emmet-Booth, C. Gonzalez Fischer, M. Herrero, M. Howden, and S. Leahy. 2021. “How Necessary and Feasible Are Reductions of Methane Emissions from Livestock to Support Stringent Temperature Goals?” *Philosophical Transactions of the Royal Society A* 379 (2210): 20200452. 10.1098/rsta.2020.0452. PMC8480228 34565223

[cit0015] Smith, P., J. Adams, D. J. Beerling, T. Beringer, K. V. Calvin, S. Fuss, B. Griscom, et al. 2019. “Land-Management Options for Greenhouse Gas Removal and Their Impacts on Ecosystem Services and the Sustainable Development Goals.” *Annual Review of Environment and Resources* 44 (1): 255–286. 10.1146/annurev-environ-101718-033129

[cit0016] Többen, J. R., M. Distelkamp, B. Stöver, S. Reuschel, L. Ahmann, and C. Lutz. 2022. “Global Land Use Impacts of Bioeconomy: An Econometric Input–Output Approach.” *Sustainability* 14 (4): 1976. 10.3390/su14041976.

